# Biodegradable Biobased Polymers: A Review of the State of the Art, Challenges, and Future Directions

**DOI:** 10.3390/polym16162262

**Published:** 2024-08-09

**Authors:** Swarn Jha, Bhargav Akula, Hannah Enyioma, Megan Novak, Vansh Amin, Hong Liang

**Affiliations:** 1 J. Mike Walker ‘66 Department of Mechanical Engineering, Texas A&M University, College Station, TX 77843-3123, USA; 2 Artie McFerrin Department of Chemical Engineering, Texas A&M University, College Station, TX 77843-3123, USA; 3 Department of Electrical Engineering, Texas A&M University, College Station, TX 77843-3123, USA

**Keywords:** biodegradable biobased polymer, biodegradation, environment, life-cycle assessment

## Abstract

Biodegradable biobased polymers derived from biomass (such as plant, animal, marine, or forestry material) show promise in replacing conventional petrochemical polymers. Research and development have been conducted for decades on potential biodegradable biobased polymers such as polylactic acid (PLA), polyhydroxyalkanoates (PHAs), and succinate polymers. These materials have been evaluated for practicality, cost, and production capabilities as limiting factors in commercialization; however, challenges, such as the environmental limitations on the biodegradation rates for biodegradable biobased polymer, need to be addressed. This review provides a history and overview of the current development in the synthesis process and properties of biodegradable biobased polymers, along with a techno-commercial analysis and discussion on the environmental impacts of biodegradable biobased polymers. Specifically, the techno-commercial analysis focuses on the commercial potential, financial assessment, and life-cycle assessment of these materials, as well as government initiatives to facilitate the transition towards biodegradable biobased polymers. Lastly, the environmental assessment focuses on the current challenges with biodegradation and methods of improving the recycling process and reusability of biodegradable biobased polymers.

## 1. Introduction

**Figure 1:**
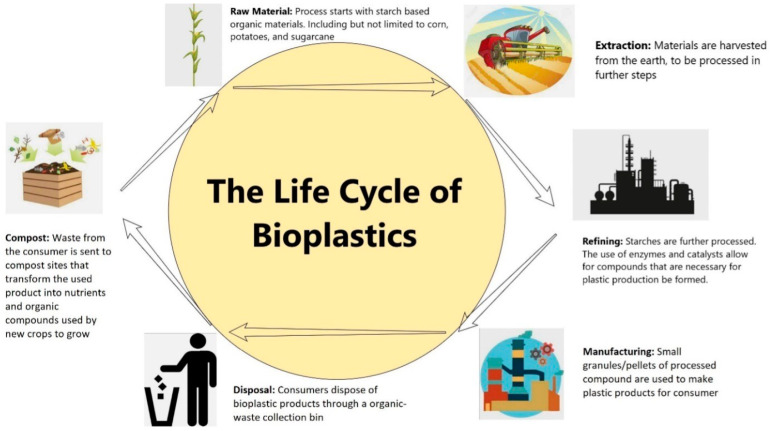
Life cycle of bioplastics (Figure recreated using sketch.io based on Figure 3 in Ref. [1]).

Biodegradable biobased polymers (more commonly known as biodegradable bioplastics) are substances entirely or partially derived from biomass or biological products, including plant, animal, or forestry materials. The advent of biobased polymers that are both biodegradable and renewable provides a green alternative to petroleum-based plastics; however, there are limitations and concerns with the current life cycle of biodegradable biobased polymers that need to be addressed before widespread adoption. Figure 1 below illustrates the general life cycle of biodegradable biobased polymers.

Some major industries that use biodegradable biobased polymers are pulp and paper, agricultural, beverage, and medical supply manufacturers [2]. Specifically, within the medical industry, poly-(*ε*-caprolactone) (PCL) is primarily used for implantable composites, bone fixation, and medicine release systems [3]. Furthermore, certain types of polyhydroxyalkanoates (PHAs) have also been shown to have applications within the medical industry. For instance, poly(4-hydroxybutyrate) (P(4HB)) was first used in 2007 to create “TephaFLEX”, a type of absorbable suture [4]. Research into P(4HB) showed that the polymer can cross the blood–brain barrier, leading to further development of using PHAs to develop various medical devices [4]. Cellophane (a cellulose-based biopolymer) is a common material used in food packaging [5]. Other polymers of interest in packaging are Polylactic acid (PLA) and Poly (3-hydroxybutyrate) [5].

**Figure 2:**
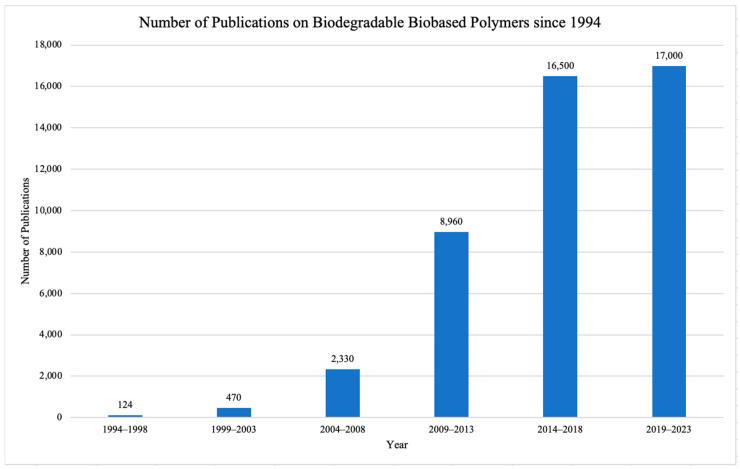
Comparison of the number of publications on biodegradable biobased polymers [obtained from Google Scholar using keyword “biodegradable biobased polymers”].

The first considerable interest in biobased polymers was during the mid-1800s when celluloid was accidentally discovered to be a thin, flexible film that could be molded into shapes and was relatively resilient [6]. In 1888, microbiologist Martinus Willem Beijerinck observed light-refractive inclusions in microorganisms, which were determined to be polymer granules [7]. Later, in the 1900s, French chemist Maurice Lemoigne discovered the ability of Bacillus megaterium to produce an intracellular polyester called poly(3-hydroxybutyrate) (PHB), from which current research has found over 100 different types of polyhydroxyalkanoates (PHAs) [8]. However, the onset of World War II stifled research into biobased polymers as society recognized the cost-effectiveness of utilizing petroleum to produce plastics. After the war era ended, research into bioplastics gradually returned. For instance, PLA emerged as another possibility for mass produced bioplastics as it could be derived naturally; however, mass production was cost- and energy-intensive [9]. As different alternatives were discovered and developed further, each material seemed to have particular use cases, and experimentation beginning in the late 1980s proved fruitful. The mid-20th century also saw the beginnings of research around degradation methods of petro-based and bio-based polymers. An example is the isolation of a PLA-producing enzyme, a thiolase, which can be used to degrade PHAs [8]. The consequence of climate change and plastic pollution [2,10,11] has continued to stimulate growth in research into the 21st century, as shown in Figure 2.

This review paper aims to provide a comprehensive overview of the current research landscape concerning biodegradable biobased polymers. It will delve into the advancements in synthesis methods and material properties, offering a detailed analysis of the technological and commercial aspects of biodegradable biobased polymers currently employed in various industries, as well as those that are undergoing active research and development. Additionally, the paper will evaluate the potential end-of-life options for these materials, contributing to a holistic understanding of their environmental impact and sustainability.

## 2. Nomenclature Used in Review Paper

Due to the confusion around jargon within the biobased polymers and biodegradability field, this section will provide definitions of technical terms used within the rest of the review paper, as discussed by the IUPAC document released in 2012 [12].

1. Biobased: Defined as completely or partially composed of or derived from biomass. This can include sources such as animal, plant, or forestry materials [12].
2. Biobased polymer: This is a polymer-derived biomass based on monomers derived from biomass. Furthermore, it can be shaped by flow throughout the various processing stages. The IUPAC organization recommends utilizing the term “Biobased polymer” over the term “bioplastic” [12].
3. Biodegradability: Defined as a substance capable of breaking down and degrading through biological activity [12].
4. Biodegradable: Represents macromolecules and/or polymeric substances susceptible to biological degradation [12].
5. Biodegradation: Represents degradation due to a cell-related phenomenon. This excludes the in vitro activity of enzymes separated from their natural environments [12].

## 3. Current State and Improvements on the Synthesis of Biodegradable Biobased Polymers

### 3.1. Green Chemistry Principles

Research focused on the synthesis of biodegradable biobased polymers has primarily focused on reducing their environmental impact by implementing the principle of green chemistry. The key principles of green chemistry are the following [13]:

- Developing circular processes.
- Using renewable feedstock.
- Using benign chemical reactants to develop benign chemical products.
- Performing reactions with a catalyst that contains abundant metals, enzymes, photons, or electrons.
- The products and reactants have weak and non-covalent interactions.
- The products and reactants are recyclable, inert, abundant, easily separable, and have low toxicity.
- Products separate from the reaction mixture without requiring additional energy or materials.
- Atom, step, and solvent economical processes.
- End-of-life maintenance.
- Proper molecule design to lower environmental impact throughout the life cycle
- Minimizing hazards and maximizing function to improve performance.
- Reduce required fresh feedstock to increase profit.

The following subsections will discuss the advancements made in the synthesis processes for biodegradable biobased polymers currently studied in academia and used in industry. The discussion of the synthesis process is broken down into the sourcing of the feedstock, the development of the biobased monomer, and lastly, the polymerization techniques to produce the final product. Within each subsection, there will be a discussion on the improvements and advancements and how they relate to the principles of green chemistry.

### 3.2. Biomass Sources

The sourcing of feedstock material is crucial in the synthesis process as it influences the properties of the end product. Plants, such as curaua, pineapple, sisal, and jute, are common sources of precursor materials due to their lignocellulosic fibers and cellulose esters [14]. Fibers extracted from these plants help enhance the mechanical strength of the final biodegradable biobased polymer [14,15]. Additionally, agricultural waste, including post-harvest waste and by-products of food processing, especially vegetable-based agricultural waste, can be utilized to produce both biodegradable biobased polymers and plasticizers, which are used to improve the pliability of a polymer [14,15].

While plant and agricultural waste are commonly used to produce first-generation biodegradable biobased polymers, research into second- and third-generation biodegradable biobased polymers has focused on feedstock material that would be viable to produce these types of polymers. Second- and third-generation biodegradable biobased polymers are important, as they help improve economic viability by extending the product life cycle, getting us closer to a circular economy. Some precursor materials used to develop second-generation biodegradable biobased polymers include non-food crops and by-products from first-generation feedstock, such as corn stover, sugarcane bagasse, wood, palm fruit brunches, and switch grass [16]. Some examples of feedstock material used for third-generation biodegradable biobased polymers include biomass from algae or industrial and municipal waste [16].

### 3.3. Biobased Monomers

Once the feedstock materials are obtained, the biobased monomers are synthesized. Significant advancements in the sustainable synthesis of biobased monomers have occurred over the past decade. The upcoming subsections will delve into two techniques for producing biobased monomers.

#### 3.3.1. Continuous Flow Ozonolysis

**Figure 3:**
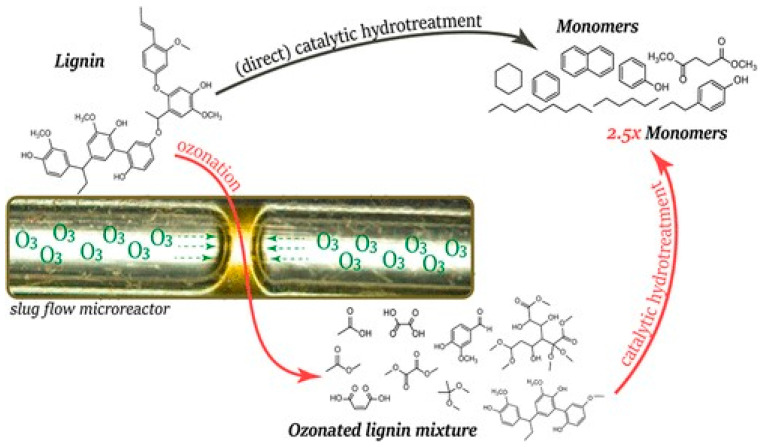
General ozonolysis reaction process. Reproduced from [17] under a CC-BY-NC-ND license. © 2019 by American Chemical Society.

The continuous flow ozonolysis process utilizes cardanol to undergo an ozonolysis reaction, where O_3_ molecules break double and triple bonds in alkenes and alkynes, producing unique monomers. In a study by Figueirêdo et al. [17], vanillin, pyrolytic lignin, and organosolv lignin were used in an ozonolysis reaction within a slug flow microreactor. The reaction took place under ambient conditions with a residence time of 12–24 s [17]. The study found that the product stream contained (di)carboxylic acids, methyl esters, and acetals. Additionally, the depolymerization efficiency was 30% for pyrolytic lignin and 70% for organosolv lignin [17]. Figure 3 showcases the ozonolysis process. This reaction process is sustainable as residual ozone quickly decomposes into O_2_, reducing the need for downstream separation processes to remove the residual ozone [17].

#### 3.3.2. Fermentation

Another method of biobased monomer synthesis is through fermentation. Recent research on fermentation has focused on improving the efficiency of the reactions, which would help lower its environmental impact. These improvements include improving enzyme performance and reducing energy consumption.

One effective method for enhancing enzyme performance is to use heterologous enzymes, which are produced by a host organism different from the one in which these enzymes naturally occur. For example, in a study, malate dehydrogenase (MDH) from Corynebacterium glutamicum was introduced into engineered Mannheimia succiniciproducens, resulting in a significant increase in the production of succinic acid to 134.25 g/L [18,19]. Additionally, promoting enzyme folding can also enhance their properties. In one study, the introduction of zwitterionic peptides to lysine decarboxylase resulted in the engineered enzyme exhibiting double the enzyme activity compared to the wild-type enzyme [18,20]. Moreover, improving protein expression levels can enhance metabolic efficiency by removing bottlenecks and regulating metabolite distribution. Furthermore, enzyme recycling can be a cost-effective method for improving enzyme performance [18]. Immobilization, for example, helps maintain the catalytic activity of enzymes even under extreme environmental conditions [18].

A method of reducing the pathway’s energy consumption is by fine-tuning hybrid pathways. A study utilizing this method found that phosphoketolase-mediated non-oxidative glycolysis enabled better accommodation of NAD(P)H/acetyl-CoA and led to a 113% yield of the theoretical maximum of 1,3-butanediol [18,21]. Another way of reducing the pathway’s energy consumption is by simply replacing the high energy consumption pathways. Researchers found a significant increase in succinic acid production when replacing phosphoenolpyruvate-dependent dihydroxyacetone kinase with ATP-dependent dihydroxyacetone kinase in E. coli [18,21].

### 3.4. Polymerization Techniques

After synthesizing the biobased monomers, polymerization techniques are employed to create biodegradable biobased polymers. The following subsections will delve into the advancements made in some promising polymerization techniques, shedding light on their benefits in the development of sustainable materials.

#### 3.4.1. Ring-Opening Polymerization (ROP)

Ring-opening polymerization (ROP) is a technique used to produce polymers from cyclic monomers. Although this polymerization technique is used in industry, there have been recent advancements made to the technique. One study by Naz et al. [22] found improvement in the polymerization process of cyclic esters by using heterogeneous catalysts. Heterogenous catalysts have the advantage of being more recyclable, having easier separation from the product, and working with a wider variety of monomers [22]. Specifically, the research discusses how MOFs (metal-oxide frameworks), which are a type of heterogenous catalyst that are beneficial as the interior and exterior of the catalyst can be used for catalytic polymerization, and their insolubility enables them to be reused without a significant reduction in the catalytic activity [22]. Some examples of MOFs that can be used in ring-opening polymerization are Ti-MOF, Zn-MOF, MDABCO MOFs, ZIF, ZnO/NC, and Co/NCF [22].

#### 3.4.2. Controlled Radical Polymerization (CRP)

Controlled radical polymerization (CRP), also known as reversible-deactivation polymerization, has rapidly developed in recent years [23]. CRP offers the advantage of producing well-defined polymers with controlled molecular weights, functional chain ends, and morphology. However, the process has limitations, such as low catalyst efficiency and chemo-selectivity. A study by Chen et al. [23] developed a novel catalyst using the side-arm strategy. Furthermore, the study found that using DMF, DMSO, or DMAC solvents in the polymerization process increased the conversion [23]. Additionally, the researchers found that using near-infrared light helped initiate polymerization and allowed for better control over the molecular weight distribution [23]. These adjustments resulted in high stereotacticity, chemoselectivity, and ultrahigh molecular weight polymers [23].

## 4. Current State and Advancements on Material Properties

### 4.1. Mechanical Properties

One important property of biodegradable biobased polymers is tensile strength. Brittle plastics, especially biodegradable ones, would lose strength in long-term storage uses and thus be less resourceful [24]. For instance, biodegradable biobased polymers derived from starch contain low water barrier characteristics and poor mechanical performance compared to traditional petro-based polymers [25]. Current research on starch-derived biodegradable biobased polymers have investigated metal-oxide nanoparticles (such as zinc oxide, silicon dioxide, titanium dioxide, and calcium carbonate), as they have favorable interfacial interactions with the biopolymer, thus improving their mechanical properties [25,26,27,28,29,30,31]. Furthermore, the metal oxide nanoparticles can block UV radiation, which similarly improves the biopolymer’s mechanical properties [25]. Unlike starch, research has shown that chitin-based plastics show impressive statistics regarding their mechanical abilities. Chitin is a major constituent in the exoskeleton of arthropods and the cell walls of fungi and is a derivative of glucose. Its natural application as a structural material is the cause for its high tensile strength, as it needs to withstand external forces [32,33]. Furthermore, improvements in the mechanical properties of free radical biodegradable biobased polymers have been observed by adding maleic anhydride, a common chemical used to synthesize unsaturated polyester resins [34,35,36]. A study that analyzed the mechanical strength of resin with varying weight fractions of maleic anhydride, n-butyl methacrylate, divinyl benzene, conjugated soybean oil, and conjugated linseed oil showed that adding a 5 wt% or 10 wt% of maleic anhydride increased the tensile strength of the resin, thus proving its use as a compatibilizer for polymers [36,37]. The mechanical strength improvement comes from maleic anhydride’s ability to enable cross-linking within the polymer. The research also showed that cationic biobased polymers exhibited higher tensile strength from adding maleic anhydride [34,35,36].

Similarly to tensile strength, elongation is another important property of biodegradable biobased polymers, especially within the packaging industry (for material suitability, stress performance, and quality control) [38]. The elongation property of materials focuses on the ability of a material to deform or stretch before breakage. Research on improving the elongation properties of biodegradable biobased polymers has focused on starch and gluten-based polymers. For instance, the branched structure observed in amylopectin can control the elongation properties of the biopolymer by improving its mechanical strength [39]. Similarly, the linear structure of amylose and the hydrogen-bonding intermolecular forces influence the elasticity of the biopolymer [25]. Similarly, gluten-based biopolymers have shown favorable elongation properties due to gliadin and glutenin, the proteins within gluten. Specifically, glutenin molecules form curly chains that bond to each other, thus developing elasticity, while gliadin is used to maintain shape in the presence of water [40]. Similar to improving the tensile strength of biodegradable biobased polymers, research into metal oxide nanoparticles has proven to also improve the elasticity and elongation properties of biodegradable biobased polymers [25].

Though PLA has already gained popularity in many practical applications, its inherent brittleness and rigidity prevents it from broader application in commercial production. Barkhad et al. sought to improve PLA samples prepared by melt extrusion and injection molding, by elongating the annealing process [41]. They observed increased compressive strength from 58 MPa for their fast-cooled PLA sample to 108 MPa for the PLA sample they allowed to anneal for 24 h, an overall improvement of 84% [41]. A 73% improvement in the Young’s modulus was also observed in the 24-hour-annealed PLA as well, reaching a maximum of about 3000 MPa, compared to the fast-cooled PLA’s Young’s modulus of 1700 MPa [41]. These improvements in rigidity and strength are directly related to increased crystallization allowed when the annealing time is lengthened.

PHB is also a generally brittle and rigid polymer. In modulus and elongation examinations performed on biobased and petro-based polymers, de Beukalaer et al. found that PHB had a Young’s modulus of 3510 MPa and a tensile strength of 43.9 MPa [42]. Impressively, the PHB material’s Young’s modulus is over 3x more than the petro-based HDPE sample’s of 924 MPa [42]. However, the PHB proved to be incredibly rigid, breaking at an elongation percentage of 1.6%, nowhere near the HDPE sample’s 617% [42].

To combat the brittleness of PLA and the lack of elongation of PHB, Armentano et al. synthesized a PLA–PHB copolymer, homogeneously dispersed with an oligomer of the lactic acid [43]. The polymer blend was synthesized using 15% wt of PHB resulting in a PLA_15PHB sample [43]. Carvacol (10% wt), a natural antibacterial agent, was also added to the blend, resulting in a PLA_15PHB_10Carv sample [43]. The group observed an elastic modulus of 1220 ± 140 MPa and elongation at break of 100% ± 40 for PLA_15PHB and an elastic modulus of 1130 ± 160 MPa and elongation at break of 105% ± 26 [43]. The neat PLA sample had a modulus of 1300 ± 180 MPa and an elongation at break of 90% ± 30 [43]. By adding 15%wt PHB, and later 10% wt carvacol, the polymer blend samples decreased slightly in elastic modulus, but increased slightly in elongation at break [43]. For films and food packaging, creating a more flexible biobased blend while maintaining strength and stability is an encouraging achievement for the future for biodegradable biobased polymers in the preservation packaging.

### 4.2. Thermal Properties

The thermal properties of biodegradable bioplastics have also been an area of focus due to their applications in packaging, shock absorption, heat preservation, energy preservation, and building insulation [44]. Specifically, cellulose, pectin, chitin, chitosan, and starch have been used due to their low density, porosity, and thermal conductivity [45]. Although current processing methods involve energy-intense processes such as supercritical drying, current research looks at ways to conserve energy and utilize sustainable and low-cost materials [44,45]. For instance, chitosan aerogels developed from freeze casting exhibited fire resistance and thermal insulation [45,46]. This is because freeze casting can obtain anisotropic porous materials with better thermal management [45]. Similarly, research into energy-efficient freeze drying and magnetically assisted squeezing and ambient drying of chitosan biopolymer foams also exhibited low thermal conductivity, thermal stability at 220 °C, and much better fire resistance than common petroleum-based materials [44].

PLA is quite malleable, especially when heat is applied, which allows it to be extruded, injection-molded, and otherwise manipulated into different shapes. These possibilities are best represented by PLA’s dominant use as a filament for 3D printing. However, PLA has low thermal stability, which prohibits its use in certain applications [41,43]. To widen the scope of possible PLA applications, methods of synthesizing thermally stable PLA must be explored. Barkhad et al. found that increasing the annealing time of their PLA sample to 24 h allowed for increased crystallization, and subsequently, greater thermal stability and conductivity [41]. Comprehensively, thermal conductivity increased as annealing time increased. At 25 C, the 24-hour-annealed PLA sample had a thermal conductivity of 0.09044 W/(m*K), up from the neat PLA’s 0.06426 W/(m*K) [41]. In addition to the increased mechanical properties discussed in the previous subsection, increasing the annealing time of PLA could be a viable, low-cost option to improve the physical properties of PLA, as long as large-scale production can reliably implement the method of synthesis.

In de Beukalaer et al.’s study, PLA had a melting temperature of 145 °C and a heat distortion temperature of 53.9 °C ± 0.4 [42]. In comparison, PHB had a melting temperature of 182 °C and a heat distortion temperature of 138.4 °C ± 0.4 [42]. Not only are the thermal properties of PHB impressive when compared to PLA, but they are still impressive when compared to some petroplastics like HDPE, which obtained a melting temperature of 132 °C and heat distortion temperature of 60.2 °C ± 0.1 [42]. PHB has truly impressive thermal properties, but is still limited in application due to its mechanical properties, as discussed in the previous subsection.

PEF, like PHB, also possesses impressive thermal properties, even when compared to its petroplastic counterpart, PET. In a work conducted by Burgess et al., the PEF sample obtained a melting temperature of 247 °C and a thermal degradation onset temperature of 413 °C [47]. The PEF sample obtained a melting temperature of 211 °C and a thermal degradation onset temperature of 389 °C [47]. Compared to the melting temperatures and heat distortion temperatures of PLA, PEF proves to be far more thermally stable and reliable. As the popularity of PEF climbs, and large-scale production becomes feasible, its thermal properties and similarity in structure to PET will establish it as a versatile biopolymer.

### 4.3. Barrier Properties

Use of biobased biodegradable plastics are contingent on favorable barrier properties. Unfortunately, compared to their petroplastic counterparts, biodegradable polymers exhibit poor gas and water barrier performance, which restricts their usage in food packaging [48]. Only a few biodegradable polymers have been able to meet the barrier requirements for certain packaging requirements. Specifically for food packaging, good gas and moisture barrier performance is necessary. For food packaging plastics with good water barrier performance over gas barrier performance, the plastic industry has typically relied on petro-based low-density polyethylene (LDPE), high-density polyethylene (HDPE), and linear low-density polyethylene (LLDPE) [49]. These plastics range from 0.91 to 0.94 g/cm^3^, 0.94 to 0.97 g/cm^3^, and 0.916 to 0.940 g/cm^3^, respectively [49]. LDPE was shown to have a low water vapor transmission rate (WVTR) of 0.2–0.4 nmol/m*s/day at 90% relative humidity (rh) and 38 °C, but a high gas permeability of 500–700 nmol/m*s*GPa at 23 °C [49,50,51]. These petroplastics, at these densities, have good processibilities to be used as plastic bags and bottles, as well as good water barrier performance [49]. In order for biobased biodegradable plastics to be used as reliably as petroplastics in food packaging, their gas and moisture barrier properties have to be comparable to their petroplastic counterparts.

PHB appears to be a better alternative than PLA for films in food packaging. In experiments conducted by de Beukelaer et al., they found that PLA had a WVTR of 35.5 ± 1.0 g/m^2^.day at 85% rh and 23 °C and an oxygen transmission rate (OTR) of 155 ± 2 mlO_2_/m^2^.day.bar [42]. PHB had a WVTR of 5.5 ± 0.1 g/m^2^.day at 85% rh and 23 °C and an OTR of 23 ± 3 mlO_2_/m^2^.day.bar [42]. In the same study, the group found their sample of HDPE to exhibit a WTVR of 0.9 ± 0.1 g/m^2^.day at 85% rh and 23 °C and an OTR of 696 ± 37 mlO_2_/m^2^.day.bar [42]. Based on the usage of HDPE for blow-molding applications in food product packaging and household product packaging, PHB seems to be a promising biobased biodegradable alternative to HDPE. HDPE had a far more advantageous WVTR, but PHB had a more favorable OTR for food packaging than HDPE.

On the newer end of biobased biodegradable polymers, great interest has been given to poly(ethylene 2,5-furandicarboxylate) (PEF) due to its enhanced barrier properties. Even compared to its petro-based counterpart, poly(ethylene terephthalate) (PET), it is shown to be an up to 31× more effective barrier to CO_2_ [52,53,54]. In experiments conducted by Burgess et al., they found PEF to be a more effective water and oxygen barrier than PET. At 35 °C, the O_2_ permeability of amorphous PEF was up to 11× lower than PET, while the water diffusion of PEF was up to 5× lower than PET [55,56,57,58]. PEF is thought to show these good barrier qualities because of the presence of furan rings, the furan ring’s high polarity and, subsequently, the difficulty of those furan rings to flip [58,59,60]. These excellent barrier properties show that PEF is one of the most optimal bioplastics available to possibly replace PET in bottle and packaging production.

## 5. Techno-Commercial Analysis

### 5.1. Introduction to Techno-Commercial Analysis

Biodegradable biobased polymers are impactful in the environmental view of plastic production and plastic waste management, but also the techno-commercial view, as well as the global economic view. In this section, the impact, current and future, of biodegradable biobased polymers is discussed in the context of a case study analysis of one country, global market trends, government initiatives, and life-cycle analysis.

### 5.2. Financial Assessment Using Case Study of PLA

Below is a case study of PLA production in Thailand. This study will analyze the cost–benefit of switching to biopolymers. It will compare different scenarios to examine the economic costs and benefits of PLA production.

#### 5.2.1. Cost–Benefit Analysis of PLA Production

In Thailand, high-density polyethylene (HDPE) production costs were calculated by determining the direct and indirect costs. Specifically, the direct costs observed were the production costs as well as the investment costs. The production cost was calculated by taking the sum of the total cash cost and the depreciation (depreciation rate of 4%) associated with HDPE [61]. For the indirect costs, the paper analyzes the costs associated with environmental emissions and the land’s opportunity cost. Specifically, the total indirect cost is the sum of the cost of carbon dioxide, methane, carbon monoxide, sulfur dioxide, and nitrogen dioxide emissions. The paper states a total cost of 329.66 million USD per 100,000 tonnes of HDPE, with the total direct costs coming to 185.74 million USD per 100,000 tonnes of HDPE [61]. Many indirect costs were attributed to the carbon dioxide and methane emissions, resulting in a cost of 124 and 13 million USD per 100,000 tonnes of HDPE, respectively [61].

Although various precursor materials can be used to produce PLA, PLA produced from cassava root was used for this analysis, as Thailand was ranked 4th in the world for cassava production and was the highest exporter in the global markets in 2007 [61,62]. Two cases were analyzed, with the first case focusing on using cassava root to produce the PLA, while the second case focused on purchasing the cassava starch directly for PLA production. For the first case, the total production cost of PLA was determined using Equation (1), as shown below [61]. For the second case, Equation (2) is used [61].

Total production cost of PLA from cassava = Total production cost of PLA from corn

− production price of corn − production price of corn starch + production price of cassava starch(1)

Total production cost of PLA from cassava = Total cost of PLA from corn

− price of corn starch + price of cassava starch(2)

Because converting the sugars into PLA is widely recognized and commercialized, the production cost of PLA from cassava is derived from the production cost of PLA from corn [61]. The total production costs are used to calculate the total direct cost of production, which is the sum of the production costs and investment costs. The total indirect costs are the sum of associated emissions. Table 1 tabulates the total direct and indirect costs that were involved in both cases.

Comparing cases one and two (using Table 1), one can conclude that PLA production using the case one process has a higher net benefit and even a 25 times higher benefit than the HDPE process [61]. The low net benefit of case two was due to not including the benefit of selling the byproducts, as the second case starts with purchasing cassava starch [61]. One thing to note is that the net benefit of PLA is driven by the much higher selling price of PLA compared to HDPE, with the initial investment cost-minimizing the net benefit of the PLA production (for both case 1 and case 2) [61]. However, this is attributed to the fact that PLA production process technology is still in its initial stages of development. The initial investment cost will subsequently be lowered with improvements to the production process.

#### 5.2.2. NPV and Sensitivity Analysis of PLA Production

For NPV (net present value) analysis, a time range of 25 years was observed, and the second case (discussed earlier) will be used as the basis for the analysis [37]. A discount rate of 1.89% will be applied when performing the NPV analysis, which was calculated using Equation (3) below (assuming a nominal discount rate of 4.77% and inflation of 3%) [61]. Furthermore, it was assumed that the lactic acid production cost was 45% of the total PLA production cost [61]. Equation (3) was employed [61] to calculate the NPV.

(3) $$NPV=\sum_{t=0}^{n}\left(\frac{V_{t}}{{\left(1+i\right)}^{t}}\right)$$

where

*t* = Year

*i* = Discount rate 

*V_t_* = Value at time t *n* = 1, 2, …, 25

Three options were observed. Option one is the baseline analysis of the case two scenario. Option two represents the impact of the yearly reduction in the selling price of PLA. Option three focuses on the effects of technological advancements on the NPV (net present value).

**Figure 4:**
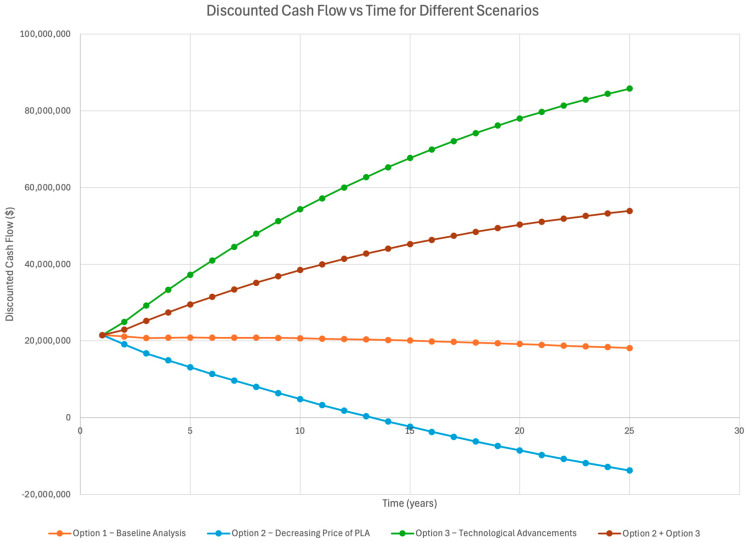
Plot of discounted cash flow vs. time (Original figure created using data from Ref. [61].)

Figure 4 below plots the yearly discounted cash flow for each case.

From the analysis of the net present value of PLA (as shown in Figure 4 above), the positive NPV value after a time horizon of 25 years proves the economic viability of the production process. The baseline analysis calculated an NPV of USD 499,566,408 [61]. Under case two, even with the 0.7% decrease in the selling price of PLA, the NPV for a 25-year time horizon came out to be USD 36,142,963 [61]. For option 2, which focused on the effects of technological advancements on the production process of PLA, it was assumed that there was a 4% reduction in lactic acid production each year [61]. With this cost reduction (and keeping the selling price of PLA constant), the NPV was calculated to be USD 1,482,543,147 [61]. An NPV analysis of combining options 2 and 3 showed a positive NPV of USD 1,019,119,702 [61]. Moreover, the analysis of the technological advancements of the PLA production showed a positive discounted cash flow over the course of the 25 years [61]. A further sensitivity analysis of the impact of the discount rate on the NPV showed that even when increasing the discount rate from 1.89% to 7.00%, the NPV starts to decrease but remains a positive value [61]. This means that the discount rate has a minimal impact on the cost and benefit of PLA production.

### 5.3. Market Trends and Demands

The push for bioplastics to take up a greater share of the plastic market is shared by both everyday people, and governments. Even so, synthetic plastics make up the vast majority of plastic production. Over 400 Mts of synthetic plastics were produced in 2022 worldwide. China alone accounted for 32% of worldwide plastic production, while North America accounted for 17% [63]. In the US, less than 6% of plastics were recycled, and unfortunately, this rate is trending downward [64]. Not only has there been a marked increase in synthetic plastic production over the last 70 years, but with the reported lack of plastic waste management, the number of non-biodegradable plastics that will end up in landfills and oceans will also see a marked increase. Borelle et al. estimated that up to 53 Mt of plastic waste will enter water-based ecosystems by 2030 [65]. If no reliable alternatives to synthetic plastics are widely adopted, global synthetic plastic production is expected to double within the next 20 years and global plastic pollution could reach 66.1 Mt/y by 2050 [66,67].

**Figure 5:**
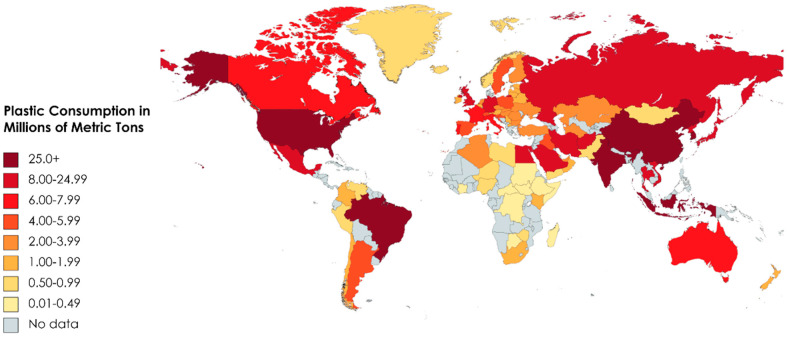
Plastic consumption by country (Original figure created using data from Ref. [70]).

Although there are economic challenges in transitioning completely towards biodegradable biobased polymers, they have been shown to contribute to a more sustainable life cycle and circular economy. Especially with the increasing demand for plastics (as shown by Figure 5 below) and the subsequent environmental and societal impacts of using plastics derived from fossil fuels, there are many benefits for transitions towards biodegradable biobased polymers. For instance, research conducted by Brizga et al. reported that there is a potential to save 241 to 316 Mt of carbon dioxide per year by substituting 65.8% of all conventional plastics [68]. Furthermore, research by Wiess and Haufe reported 55 ± 34 GJ/t and 127 ± 79 GJ/ha of energy saving from the production of biodegradable biobased polymers compared to conventional plastics [69]. From a production standpoint, this can benefit chemical-manufacturing corporations, as reduced carbon emissions and energy savings can improve the OPEX of the chemical plant (as shown in more detail in Section 5.2).

The current market for biobased polymers is projected to reach USD 29.8 billion by the year 2027 (with a CAGR of 18.2% between 2022 and 2027) [71,72]. One main factor influencing this growth is the increasing environmental concerns with the production and use of synthetic plastics derived from non-renewable sources. Due to increased consumer awareness of the negative environmental impacts of single-use plastics, demand for biodegradable biobased polymers has increased as more consumers are looking to reduce their carbon footprint [71].

#### 5.3.1. Current Market and Demands

Bioplastics, as a whole, had a market share of 0.5% in 2022 [73]. Presently, PLA is the leading biodegradable bioplastic because of its durable physical properties, diverse material sources, and mass production ability [74]. Notably, it is the most common 3D printing material for consumer use. With an increase in production from 0.2 million tons in 2015 to 0.3 million tons in 2019, PLA is the most prevalent biodegradable polymer available [75]. Its production rate and market value are only expected to increase, possibly reaching a U.S. market value of 3.2 billion USD in 2032 from 1 billion USD in 2022 [63]. However, as explored in the previous section, the cost of mass-scale production of PLA is prohibitive and a barrier to global adoption. PLA also breaks with less than 10% elongation, indicating its fragility and ineffectiveness in some product applications [76], namely those that need to endure high stress. Another hindrance to greater application of PLA is its low degradation rate of 3–5 years [10]. Medical and biomedical applications of PLA are not ideal because low degradation rates in the body could cause adverse reactions [76,77]. To ensure a sustainable switch from petroleum-derived synthetic plastics to PLA, or any other biobased biodegradable polymer, the cost of production must either be reduced or heavily subsidized.

Polyhydroxyalkanoate (PHA) is also a promising alternative to synthetic polymers especially as applied in food packaging [78]. It is a microbial compounding plastic, synthesized from many bacteria groups, and can be produced from a wide variety of sources, namely sucrose, corn, and vegetable oils [78,79,80]. Although PHA has a decreased thermal processibility compared to PLA, it fills the gap of biodegradable polymers in biomedicine that is left by PLA’s low degradation rate [76,81]. Poly(3-hydroxybutyrate) (PHB) is currently the most produced subset of PHA [78,79]. It has a comparable molecular structure to polypropylene (PP), and so shares some of its physical properties, such as water resistance, tensile strength, and melting point [82]. Despite the abundance of starting materials, industry-wide adoption is hindered by its high cost of production [78,81,83]. Over 50% percent of industrial production costs of PHB comes from heterotrophic fermentation of an organic carbon source [82]. Price et al. suggests producing cyanobacterial PHB using atmospheric CO_2_ rather than an organic carbon substrate required by heterotrophic fermentation could significantly reduce the cost of production of PHB [82].

Biobased poly(butylene succinate) (PBS) has been shown to be a possible replacement for petroleum-based polymers like PP and high-density polyethylene (HDPE). It can be synthesized from corn, sugar beet, sugar cane, and potato, all of which have minimal land and water requirements [84]. PBS is an aliphatic polyester derived from 1,4-butanediol, succinic acid, or dimethyl succinate, among other monomers [84,85]. Due to the increased demand on biodegradable polymers, including PBS, global succinic acid production has dramatically increased. It is estimated that the global succinic acid production through the 1990s ranged from 15,000 to 18,000 Mt annually before climbing to 30,000 Mt in 2011 and an estimated 700,000 Mt in 2020 [84,86]. It is also estimated that at a CAGR of 27.4%, the succinic acid market value will come to $1.8 billion globally by 2025 [86]. Typically used in sheet, film, and food-packaging production, PBS, like PLA and PHB, has a wide variety of applications. For PBS to have a greater impact, forcing reduced production of its petro-based analog, PBS synthesis must begin to equal the cost and ease of the petroplastics currently in production.

#### 5.3.2. Potential for Future Market and Demands

Newer biodegradable bioplastics have emerged in the industry since the late 2000s and early 2010s. Poly(ethylene 2,5-furandicarboxylate) (PEF), synthesized from FDCA and ethylene glycol (EG), is a polymer rising in popularity due to its similarities to PET [52]. Not only is it similar to PET, but it also exhibits more favorable barrier properties, such as gas and water permeability, than PET [55,56,57,58]. First patented in 1946 by Celanese Corporation, interest in PEF took the backseat in popularity to more easily mass-produced biobased plastics like PLA and PHA [87]. But starting in 2009, works from Gandini et al., Gomes et al., and Jiang et al. generated massive interest in PEF synthesis and prompted efforts towards the low-cost mass production of PEF [88,89,90]. Currently, Avantium is working to broaden the commercialization of PEF using their YXY Technology, with the goal of replacing many PET-dependent products, like bottles, industrial fibers, and clothing textiles, with PEF [91,92,93]. Such a task is daunting, but with the favorable properties of PEF, the potential to replace not only PET, but other materials like glass and aluminum, in various industries from packaging to textiles could radically alter these markets in the future.

Biobased polyamides are also being looked at to combat the increase in petroplastics. Though they are of great interest, they have not been fully realized or commercialized yet.

Promising research by Lee et al. showed the ability to synthesize biodegradable bionylon4,4 and -5,4 using succinic acid, 1,4-diaminobutane, and 1,5-diaminopentane procured from microorganism fermentation [94]. The group observed excellent water absorptivity as the bionylon -4,4 sample exhibited absorptivity of 18.0 ± 0.3 uptake %, while the bionylon-5,4 sample exhibited absorptivity of 19.6 ± 0.2 uptake % [94]. For comparison, petro-based nylon6,6 in the literature has shown a water absorption range of 2.05–10% [95,96,97]. The water absorption performance of these bionylon samples are promising and could be highly effective in nylon films and products requiring high wettability [94]. These samples also exhibited high melting temperatures compared to their petro-based counterpart. The bionylon-4,4 sample had a melting temperature (Tm) of 299.1 °C, and the bionylon-5,4 sample had a Tm of 275.6 °C. Comparatively, comparison, the chemnylon-6,6 sample had a Tm of 253.2 °C [94]. The high amide density of the bionylon samples allow for enhanced water absorption and thermotolerance [94]. Combined with their biodegradability, bionylons are a promising replacement for petro-based nylons.

### 5.4. Commercial Impact of Technological Innovations

The commercial impact of advancements in biobased biodegradable polymer technology has potential to be disruptive. The capability for commercial impact is first apparent in the cost of synthetic plastic production and plastic waste management. Between 40% and 50% of all plastic produced is single-use plastic, used for product packaging. If biobased biodegradable plastics can reliably replace this subset of plastic production in the near future, the threat of environmental stress from landfills and water contamination can be greatly reduced. A reduction in environmental stress, and the government taking on the responsibility of handling plastic waste, could result in resources otherwise allocated for waste management, to be reallocated to more socially beneficial programs. An estimated 14.5 Mt of plastic waste was released into the ocean in 2018 and is projected to multiply 2.6× by 2040 if the plastic production and consumption rate does not decrease [98,99]. Plastic waste management alone is a 34 billion dollar market and is only expected to grow given current plastic production and waste rates [100]. The global plastic industry is valued at a massive 700 billion USD, as plastics are heavily integrated in numerous other industries. In order for biodegradable polymers to have a meaningful commercial impact, its promotion from 0.5% of plastic production to replacing the subset of plastic production used for packaging is paramount [73].

Current commercial impact, as mentioned in preceding sections, is seen not only in the product-packaging industry, but also the medical and biomedical fields. From surgical applications, drug delivery, and medical implants, biodegradable polymers have a 50-year history of impact and improvements in the medical industry [101]. PLA and PGA lead this front in suture materials due to their dependable flexibility, while PHAs are relied on for intrabody procedures due to their water insolubility and biocompatibility [102]. The biodegradable biomaterials market has seemingly grown faster than the bioplastics space because of the clear benefits to using biobased materials in the body over non-biobased materials [103]. The gap that needs to be filled in this space deals with greater coverage of intrabody materials. Metal plates, rods, and screws are still standard over their biobased counterparts because of their structural stability and reliability. Improving structural rigidity and dependability of biobased biomaterials can push more sustainable medical solutions.

The advancements made in established biobased biodegradable polymers such as PLA and PHA have already had a significant impact since their commercial introduction in the 1990s. It is apparent though, that in order for these and future innovations to have a greater socio-economic impact, the cost of production must decrease. Methods of production that do not require virgin raw material would also greatly improve the feasibility of socio-economic impact, mainly reducing waste management costs, reducing synthetic waste production, and halting environmental harm from plastic waste. Researchers, however, are still developing and improving in the field of biodegradable biobased polymers and are showing promising results for the future of synthetic plastic replacement.

### 5.5. Government Initiatives towards Biodegradable Biobased Polymers and the Commercial Impact

Policy is proven to be key for bioplastic success. Morone et al. [104] used Social Network Analysis (SNA) to investigate the ties and relations between producers, suppliers, and institutions in the Italian bioplastics network. Research, as such, has applications in policymaking, as these investigations can be used to guide lawmakers who can stimulate collaborative behaviors. Unexploited potential exists in terms of interactions within the niche, and a more efficient use of resources can be employed.

Plastic waste is the third-highest contributor to solid waste in cities. Legislation can redirect and incentivize alternative disposal methods. Instead of the traditional landfill, as the economy shifts to bioplastics, waste disposal efforts could be directed toward developing efficient composting sites and the onset of new technologies that could sort bioplastics to effectively utilize waste. Increasingly, governments are growing aware of the mounting plastic waste and the environmental hazards they impose, opting to ban single-use plastics and plastics made of non-biodegradable material. However, banning these materials and products is only a start; replacing those materials and products with those made of biodegradable polymers is also a necessary step to significantly decreasing the amount of environmentally hazardous waste.

**Figure 6:**
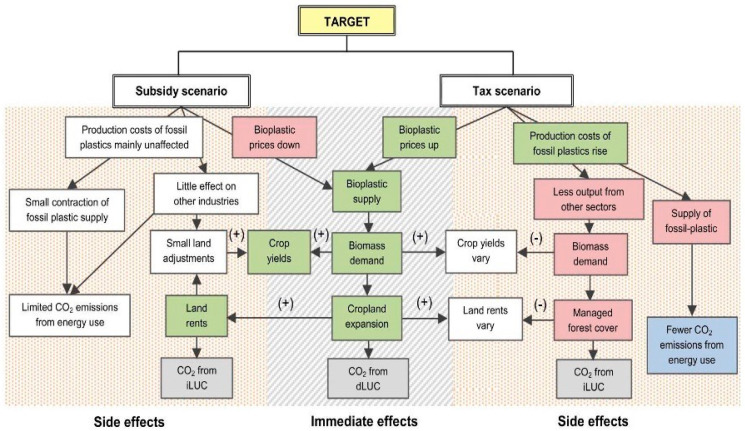
Impacts of a subsidy and tax on economic and environmental factors. Image reprinted from Ref [106] with permission under Creative Commons Attribution 3.0 license.

Currently, a few policies govern bioplastics’ growth, and around 70% of federal funding for research has gone to biofuels since the 1970s [105]. An increase in public demand for bioplastics could lead to government action that could effectively cause private partnerships for building facilities and research on bioplastics-based infrastructure. A high entry cost into the market is what is stopping manufacturers and interested parties from being active producers, and the role of lawmakers is crucial to this technological revolution. Using a subsidy on bioplastics versus a tax on petroplastics is the focus of a work produced by Escobar et al. [106], from which a figure explains each path’s economic and environmental impact. Figure 6 shows this impact, where green (+), colorless (0), and red (-) show the respective impact of the variable; and gray (+) and blue (-) show the respective impact on CO_2_ emissions. A subsidy was calculated to increase the aggregate demand for plastics by 0.32%, whereas the tax reduces the plastic market by 7.24%, which reduces global demand for petroleum and coal products and has spillover effects on the agriculture and food production industries [106]. The impact of the tax overall was much more far-reaching than that of the subsidy despite increasing bioplastic production by the same amount. As the bioplastics market is relatively small, a subsidy does not change nearly as much. It only notably increases the demand for intermediate feedstock. The side effects of taxes on conventional plastics include factor reallocation and a large contraction in the plastics industry, which affects all markets that rely on plastics due to a higher input cost. Policy considerations, as such, must consider every impact that an incentive can create.

Even so, governments have been implementing regulations to reduce single-use plastics (as seen with the EU’s Single Use Plastics Directive) and increase the production and use of biobased and biodegradable polymers [71]. Such initiatives create a favorable market for biodegradable biobased polymers and motivate more sustainable practices within the chemical manufacturing industry [107,108,109].

The commercial viability of switching from conventional petroplastics is a concern of note. For instance, to replace the total amount of plastic packaging used within the EU, this would require 70.3 Mt of corn, 0.08 Mt of castor beans, and 3.1 Mt of wood [68,110]. To produce this much precursor material, one must have 7.4 million ha of land and utilize 45 billion cubic meters of water to produce the required amount of biodegradable biobased polymers [68]. Essentially, this would mean that the EU would need to allocate more than half its agricultural land (currently, we use approximately 0.02% of agricultural land for the production of precursors) and use approximately 60% more than its annual freshwater withdrawal, which is currently unfeasible [68,111]. However, there are potential solutions to this problem. One method of improving the viability is using second-generation biomass, which are non-edible biowastes [111,112]. More than 1 billion tons of agricultural and food waste are produced yearly, which can be processed using inexpensive biorefining processes [111,112]. Other methods to improve viability are improving the conversion within biorefinery processes, switching to renewable energy within biorefineries, and improving end-of-life management by recycling bioplastic wastes or composting [111,112]. Although the commercial viability of switching to biodegradable bioplastics in our petroplastic world is incredibly difficult, these solutions show that the hindrances are not insurmountable.

### 5.6. Life Cycle and Cradle-to-Grave Assessment

Analysis of the commercial potential has shown increasing consumer demand and market favorability for biodegradable biobased polymer, even if challenges must be overcome before a complete transition can occur. However, life-cycle assessments must be performed to develop an in-depth understanding of the polymer throughout its various life stages, starting from harvesting the precursor materials. Many frameworks are used to perform this analysis, with some of the most common being life cycle analysis, cradle-to-grave, and cradle-to-gate [113]. Although this analysis focuses largely on the environmental impacts, it also affects the economic costs as companies that fail to consider the environmental costs of their production processes tend to have higher regulatory compliance costs, operational efficiency, business continuity, and investor relations.

One report summarizing the life-cycle assessment provided the results of various studies that utilized the cradle-to-grave analysis method to measure the advantages and disadvantages of transitioning away from conventional petro-based plastics. Overall, the studies showed that, although biopolymer production had a higher impact on acidification, photochemical ozone formation, and eutrophication, with regard to climate change, biopolymer production had a much more positive impact than with fossil-fuel-based polymers [114]. Specifically, Harding et al. performed a cradle-to-grave analysis on the environmental impacts of PHB. They found that although the PHB had greater environmental impacts during the cultivation and the amount of water and steam needed in the process stage, the production of PHB was more environmentally friendly than the fossil-based versions, such as polypropylene (PP) and polyethylene (PE) [114,115]. Furthermore, studies by Khoo et al. and Khoo and Tan showed that from using the cradle-to-grave and cradle-to-gate assessment frameworks, utilizing renewable energy during the processing stages improved the environmental impact of PHA production [116,117,118]. Moreover, the cradle-to-grave analysis showed that the most favorable option for end-of-life is composting, while the worst disposal method is sending to landfills, which results in methane release [114].

Another common biodegradable biobased polymer is PLA. Specific studies that focused on the production of PLA found that the production phase tended to have the largest environmental impact, especially on ozone depletion and aquatic ecotoxicity [75,114]. Compared to fossil-fuel-derived polymers, PLA performed better than fossil-based PET but worse than fossil-based PS. Moreover, studies showed that the greenhouse gas emissions of PLA were higher than those of both PP and PE when the polymer was sent to landfills. However, when production was under renewable energy use, PLA production was shown to have lower environmental impacts than fossil-based PS and PP [119,120,121,122,123,124]. Furthermore, the best end-of-life scenario for PLA was determined to be composting.

Similar to studies conducted on PHB and PHA, studies on bio-PE have shown it to reduce greenhouse gas emissions, in comparison to their fossil-fuel-derived counterparts (a ratio of 5.1 kg CO_2_eq/kg PE to 1.26 kg CO_2_eq/kg bio-PE) [125]; however, the processing of bio-PE has been shown to have greater acidification and eutrophication impacts than its fossil-fuel-derived counterparts (specifically having a 1.6 to 2 times impact) [126]. Location also played a role in the life-cycle analysis of bio-PE due to differences in precursor harvesting techniques around the world [127]. A study by Posen et al. showed that sugarcane-based bio-PE produced in Brazil had lower greenhouse gas emissions than those produced in the U.S. [120].

Overall, the life-cycle assessment showed that the major environmental impacts in producing biopolymers include acidification and eutrophication. However, switching to renewable energy sources rather than water and steam greatly improves the process by reducing environmental impacts. Life-cycle assessments on land occupation and land use change showed that to transition towards biopolymer use, large amounts of land need to be used, and even a 5% increase in biopolymer consumption can reduce the favorability of biopolymers over fossil-fuel-based polymers [106]. A possible alternative to this issue is the use of second-generation biopolymers, which can be implemented by shifting to composting rather than sending biopolymers to landfills at EOL (end-of-life) [106,114].

## 6. Assessment on the End-of-Life of Biodegradable Biobased Polymers

### 6.1. Analysis of Biodegradation Rates of Biopolymers under Different Mediums

One major issue with adopting biodegradable biobased polymers is the low biodegradation rates. Biodegradation involves the use of microorganisms to break down the polymeric bonds. This process involves, first, hydrolytic oxidative enzymatic degradation to break down the large polymer chain into smaller fragments [6,128,129]. Once this has been performed, microbes break down the fragments into carbon dioxide, water, and cell biomass [6,128,129]. Although biodegradation can be a better alternative to photochemical degradation, many biopolymers that exhibit biodegradation can only degrade in specific conditions [6]. For instance, the biodegradation of PLA (polylactic acid) depends on various factors, such as its molecular weight, crystallinity, purity, temperature, pH, water permeability, and additives in the polymer. Furthermore, the medium in which the biodegradation happens can have a larger impact on the rate and degree to which biodegradation takes place [130,131]. For instance, in the review paper by Meereboer et al., PHB film degradation in natural mature soil may take up to 112 days for a 60% mass loss; however, it could take 80 days for an 82% mass loss in commercial soil [131,132,133]. The following sections will delve deeper into the different mediums of biodegradation and analyze their performance in degrading common biodegradable biopolymers.

#### 6.1.1. Compost

Composting is a method in which plastic is converted into CO_2_ and humus, which can be used as a nutrient-rich addition to the soil to help plants grow [128,134]. The CO_2_ produced is part of the biological carbon cycle and is not detrimental to the environment. An important consideration when using this method is the composition of the compost, which affects the types and amounts of microorganisms that would degrade the bioplastic. Moisture content must be kept track of and kept stable. Sarasa et al. [135] explains that the moisture content of compost may fall between 20 and 56%, and active monitoring is required to ensure necessary microbial activity. A method in which this can be measured and assessed is ASTM D5338 [136], which correlates biodegradability to the total mineralization of organic carbon (CO_2_ evolved) in the polymer [137]. Kale et al. [137] used this and other methods to compost PLA water bottles using CMR and GMR systems. Results from the CMR system show a mineralization value of 60% on the 39th day of PLA degradation, lagging slightly behind cellulose powder degradation due to the initial hydrolysis of PLA.

#### 6.1.2. Soil

Soil degradation is more feasible than water or air as the number of microorganisms and their relative diversities results in accelerated degradation [138]. The type and environment of the soil are crucial to how the plastic deteriorates, as each contains different types of microorganisms that vary in efficiency in regard to degradation [138]. The structure of the soil, such as the size of the particles, can change the amount of water content in the soil, gas diffusion, and even heat transfer, all of which are important to microbial activity. Innocenti [139] explains that fine, grainy soil will have free gas diffusion, whereas blocky clay soil would be poorly aerated but can retain heat well. In the fine soil, aerobic microorganisms such as fungi will develop, whereas the clay soil will develop facultative or microaerophilic aerobes. Different types of microorganisms lead to differences in biodegradation and breakdown of the bioplastic polymer [139]. Even within a singular geographical location, seasonal variances affect the nature of the soil composition [11].

**Figure 7:**
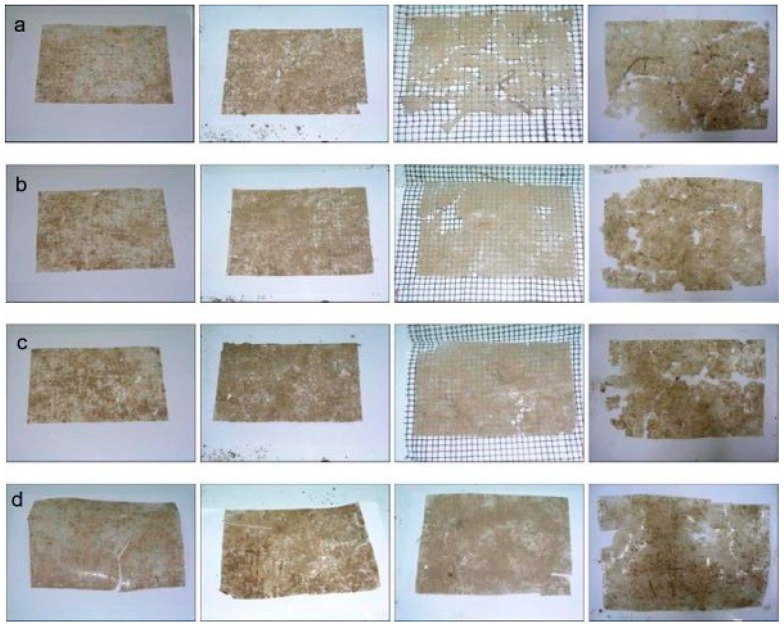
PLA (**a**) 20 mm (**b**) 40 mm (**c**) 75 mm (**d**) 400 mm thickness films at different stages of degradation. Image reprinted from Ref [140] with permission from Elsevier.

Two experiments buried in the soil but under different environmental conditions can have different results. Rudnik and Briassoulis [140] held two experiments with different PLA films (with thicknesses 20 µm to 400 µm), one in an experimental field in Spata, Greece, and the other in a bioreactor in a laboratory using soil taken from the experimental field. The laboratory experiment did not report any disintegration or mass loss for the 11-month experimental period, whereas the field experiment resulted in a gradual mass loss [140]. One similarity that the two experiments shared was an instant decrease in the mechanical strength of the PLA films, as the elongation of the films at the breakpoint rapidly decreased within the first month of the experiment. For PLA 30 and 75 (PLA with a thickness of 30 mm and 75 mm, respectively), under laboratory conditions, this measure decreased from 17.5% down to 1.3% and from 3.5% down to 1.8%, respectively, and for the outdoor experiment, the measure decreased from 6.3% to 1% for PLA with a thickness of 20 mm and from around 4.9% to 1.2% for PLA with a thickness of 50 mm [140]. This is consistent with the inert hydrolysis phase of PLA degradation [140]. The degradation is illustrated below in Figure 7.

Microorganisms in soil sensitive to pollution, heavy metals, and petroleum hydrocarbons vastly reduce the amount of microbial activity per cm^3^ of soil [76]. Biopolymers do not have nearly as much effect on the amount of bacterial mass in soil, as shown in an experiment by Adhikari et al. [141]. However, in this experiment, the diversity of bacteria throughout the degradation process did change. PLA decreased the NO_2_- oxidation activity by 26% and the NH4+ oxidation activity from 40% to 21.5%, which means that PLA negatively affected the activities of both ammonium- and nitrite-oxidizing bacteria [141].

**Figure 8:**
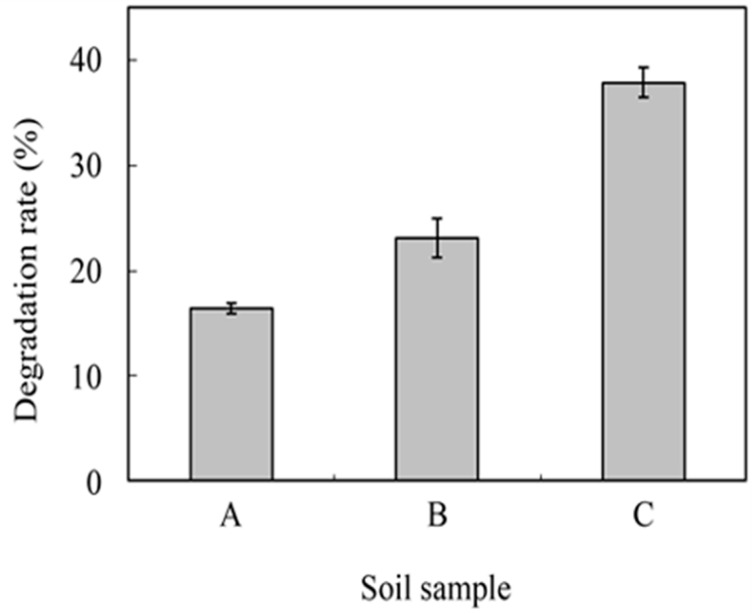
Bacterial density vs. degradation rate (A: 7.5·10^6^, B: 7.5·10^7^, C: 7.5·10^8^ cells/g-soil). Image reprinted from Ref [141] with permission from Scientific Research Publishing.

Quantifying and understanding bioplastic-degrading bacteria found in soil provides an accurate measure of the feasibility of soil degradation. Figure 8 shows the overall effect of the amount of bacterial density on degradation rates for PBS-starch plastics. Suyama et al. [142] collected three different soil samples (ando soils—37.4% H_2_O, pH 4.5; brown lowland soils 18.1% H_2_O, pH 6.0; muck soils 34.1% H_2_O, pH 5.8) and inoculated the samples onto agar plates after sterile water dilution. A total of 200 to 300 colonies appeared on 10 plates with different bioplastic/soil combinations, and the percentages of PHA, PCL, PHC (Poly (hydridocarbyne)), and PTS (poly(tetramethylene succinate))-degrading colonies were 2 to 18%, 2 to 11%, 1 to 7%, and 0 to 1%, respectively [142]. No PLA-degrading colonies were found.

Continued research into the types of bacteria that are most versatile and resilient is ongoing, and some progress has been made. Bacterium Pseudomonas chlororaphis (ZK-1) and Bacterium Cupriavidus necator (POP-31) are low-nutrient-demanding strains and are highly adaptable [141]. Research conducted by Blinková and Boturová [143] uses these two bacteria to degrade PLA, and insight into the capabilities of the bacteria is provided. Essentially, Pseudomonas is arsenic-tolerant, and Cupriavidus is isolated from an environment containing high levels of lead and antimony. Cupriavidus possesses plasmids and megaplasmids and thus can handle heavy metals and degrade organic toxic material efficiently. Pseudomonas is not nearly as researched but contains degradable plasmids that metabolize high molecular organic compounds such as polymer chains. When used to degrade PLA, these bacteria resulted in distinctive damage to the surface and structure of the PLA over 50 days. Compared to other strains, such as Bacillus cereus (ZK-27) and Kocuria rosea (SL-1), the two strains exhibited higher degradation capabilities.

#### 6.1.3. Water

Aquatic degradation of standard plastics and materials is characterized by UV-induced photodegradation [144]. The overall process is relatively slow for several reasons. Firstly, degradation is temperature-dependent, so the fact that seawater is a good absorber of heat does not help the speed of degradation [144]. Next, biological degradation is not the main component of overall degradation, as there is little oxygen for such processes. This results in plastic litter on the seafloor [145].

**Figure 9:**
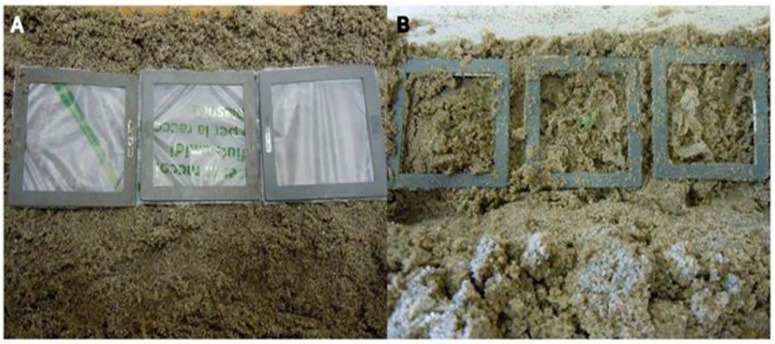
Mater-Bi Films before (**A**) and after a nine-month seawater degradation period (**B**). Image reprinted from Ref. [151] with permission from Frontiers in Microbiology.

Biodegradable biobased polymers have been researched and shown to degrade in seawater due to surface erosion via an enzyme-catalyzed hydrolysis [146,147,148,149], which, in this case, is dictated by the surface area available for enzymes to degrade [150]. An experiment by Tosin et al. [151] degraded Mater-Bi films, made of PBAT, starch, and additives, in seawater over 2 years. Figure 9 shows films of Mater-Bi before and after 9 months in seawater and sediment from the littoral zone of Marina Di Campo, Italy. One of the test methods simulated the pelagic zone of the ocean, which is characterized by low nutrient concentrations. The plastic ended up decaying heavily in terms of mechanical strength, with tensile strength decreasing 96% and elongation at break decreasing 66% during the 24 months [151]. On the other hand, LDPE, a traditional plastic used in packaging, gained mechanical strength under the same conditions.

Certain bioplastics could be more suitable for seawater/aquatic degradation than others. In terms of microbial degradation, starch-degrading microorganisms were shown to have more prevalence in 4 stations along the coast of Puerto Rico. An experiment by Imam et al. [152] used plastics made from either starch, PHBV, and a blend of both and found a rate of mass loss of 2% per day and complete decomposition in 100 days for starch, whereas PHBV had a respective rate of 0.1% per day [152]. Blends of both compounds revealed two separate biodegradation mechanisms, with starch degrading first and PHBV rapidly accelerating in decay after 50 days [152]. Degradation would lag significantly in open water, with little to no microbial activity on the plastic. A longer lag time would be experienced, as colonization onto the surface of the plastic would be much slower.

Microbial degradation is not the only pathway to degradation. Water has its own effects on the structure of a given bioplastic. Tapioca starch/PVA bioplastics are affected by water absorption, as discussed in a paper by Judawisastra et al. [153]. Starch is inherently hydrophilic and can have a maximum water absorption of 495%. After the absorption process, hydrolysis occurs, in which the C–O–C bonds are attacked, and O–H bonds are formed, which degrades starch into -D glucose monomers [153]. In this case, tweaking the amount of PVA changed the amount of water absorption and the properties of the plastic. Starch and PVA (29% by wt.) together reduced the amount of free O–H groups, which reduced the amount of water deterioration of the plastic (tensile strength decreased 30% for just starch but only 6% for PVA/starch and elongation at break decreased 36% for just starch but only 30% for PVA/starch) [153].

**Figure 10:**
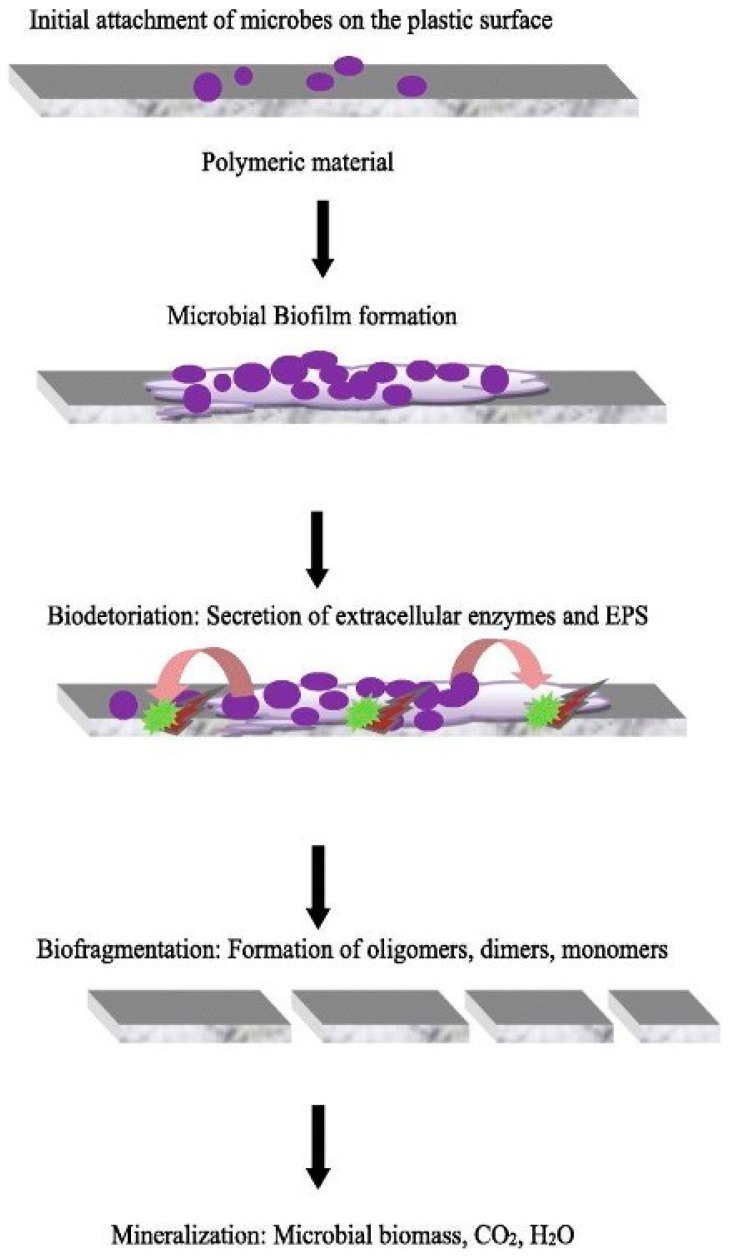
Biofilm formation to mineralization of polymeric bioplastic. Image reprinted from Ref. [156] with permission from Marine Pollution Bulletin.

Unlike soil and compost, water can be dynamic, leading to accelerated mechanical degradation of bioplastic material. Tsuji and Suzuyoshi [154] conducted an experiment where PCL, (R)-3-hydroxybutyrate, and poly(L-lactide) (PLLA) films were degraded in natural dynamic seawater at the Akabane Fishing Port in Japan. In general, compared to degradation in static water, these films degraded far more rapidly in dynamic conditions due to increased stresses and strains. All tested films showed at least a 20% increase in degradation sometime during the experiment [154]. PLLA-C films were more resistant to physical weathering as their crystalline regions act as physical cross-links and protect the films from stress generated by the moving water [154]. However, R-PHB showed rapid degradation when exposed to dynamic seawater compared to its degradation in static water, with degradation consistently being 60–70% higher in dynamic conditions [154]. Dynamic conditions, however, do not allow for biofilm production as easily as static conditions do. Increased amounts of biofilm production led to an overabundance of enzyme activity, which means more sites are active for hydrolytic degradation [131]. Deroiné et al. [155] examined the effects of biofilm on PHBV degradation and found that the addition of 5% biofilm by concentration resulted in a degradation of 97% over 200 days compared to just 36% of degradation over 180 days for natural conditions (0% biofilm concentration). The biofilm formation to the bioplastic breakdown is shown in Figure 10.

### 6.2. Advancements on Reusability of Biodegradable Biobased Polymers

#### 6.2.1. Self-Healing Polymers

Along with research on improving the degradation rates and pathways of biodegradable biobased polymers, research has also focused on improving the reusability and lifespan of these materials. One method is through innovations in self-healing polymers. Specifically, “self-healing” polymers can recover their structural functions even after damages that could compromise the material’s structural integrity [157,158]. A paper by White et al. discussed a microencapsulating healing agent that is embedded in the polymer matrix [157]. This capsule is ruptured during the formation of cracks, which releases the healing agent and initiates a polymerization reaction to fix the cracks that form [157]. The research showed a 75% recovery in the mechanical toughness [157]. Similarly, the use of Murexide salts in vinyl alcohol copolymers showed that for a mass % of between 3 and 5% of Murexide salts and a temperature greater than 60 degrees Celsius, there was a healing efficiency of over 80% [157,159]. A review paper by Wu et al. [160] looked at an alternative to hollow fiber and microencapsulation approaches (such as the paper by White et al.), which focused more on the thermal-initiated healing approaches and found that it has more potential for polymer self-healing over longer time frames.

#### 6.2.2. Memory Shape Polymers

Research has explored memory-shaped biodegradable biobased polymers, similar to self-healing polymers [161,162]. These polymers consist of non-cross-linked and reversible cross-linked polymers that can be reprocessed. The concept of dynamic polymers, first discussed in 2005, introduced the idea of supramolecular macromolecules and dynamics [163]. Covalent adaptable networks (CANs) have since expanded as a class of polymers that can combine the mechanical strength of thermosets and the moldability of thermoplastics [161,163]. CANs are characterized by dynamic covalent bonds within a network. These networks can be synthesized by polymerizing multi-functional monomers or cross-linking thermoplastics [163].

CANs can be classified as dissociative or associative. In dissociative CANs, covalent crosslinks are first broken before new crosslinks can be formed [163], whereas in associative CANs, the formation and breakage of covalent crosslinks can occur simultaneously [163,164]. Leibler et al. developed a type of associative CANs known as “vitimers”, which have a permanent cross-linked network that can be adjusted through exchange reactions [163,164,165]. For instance, a study used ozonation to introduce carboxylic acid groups into kraft lignin (KL) and then reacted it with aliphatic diepoxy monomers to form dynamic ester linkages, exhibiting vitimeric properties [163,166,167]. Other studies showed that by varying the ratio of carboxylic acid groups to epoxy, the density of dynamic ester linkages could be adjusted, allowing for a tunable material [163]. Additionally, a study developed a tri epoxy from eugenol that showed self-healing properties above the glass transition temperature [163]. These developments enable the creation of biobased polymers that can be reshaped and self-healed, thus extending their life cycle.

### 6.3. Advancements on Recycling Biodegradable Biobased Polymers

Recycling research has also been crucial in improving the life cycle of materials. Maintaining thermal stability and mechanical properties across multiple reprocessing stages poses a challenge in polymer recycling. Mechanical recycling is a recycling method that has demonstrated potential [168,169], as it has been shown to have lower non-renewable energy consumption, GWP (global warming potential), processing cost, acidification, and eutrophication [170]. According to a study conducted on the mechanical recycling of PLA, the results revealed no significant change in the Young’s modulus of the material [168]. Additionally, the tensile stress and stress at the fracture point remained largely unchanged compared to PLA without any reprocessing [168]. However, mechanical recycling of PLA has been observed to cause structural changes, as evidenced by a decrease in molecular weight. Nevertheless, studies on recycling PLA with silk nanocrystals have demonstrated a reduction in thermal degradation during melt processing [171].

In recent years, there has been expanded research into more advanced recycling methods, which includes chemical recycling and bio-recycling. Chemical recycling involves using chemical reactions to break polymers down into their individual monomers [170]. This technique can significantly improve the end-of-life (EOL) of biodegradable biobased polymers, as the monomers produced from the depolymerization process can be directly used in the creation of other products [170]. Currently, research on chemical recycling has commonly focused on PLA. For instance, a study on the hydrolysis of PLA at 250 °C showed a 90% recovery of L-Lactic Acid [170]. On the other hand, there is limited research on the performance of chemical recycling on PHAs [170]. However, studies on thermal degradation from pyrolysis and alkali–earth compound catalysts have shown to produce cis-trans crotonic acid. Crotonic acid can be polymerized to produce plasticizers, dental cement, and cosmetic products [170]. Furthermore, when PCL (polycaprolactone) is depolymerized using a methanolysis reaction, it produces methyl 6-hydroxy hexanoate. This enables a closed loop for the life cycle of PCL, as methyl 6-hydroxy hexanoate can be used to reproduce PCL [170]. Another method of recycling is bio-recycling, which employs biocatalysts, enzymes, and microorganisms to selectively depolymerize waste materials [172]. This is different from compositing or anerobic degradation, as both these methods result in the inability to reuse the end products to produce plastics [172,173]. One of the pioneering studies of bio-recycling was performed by Tournier et al., where engineering PET polymerase was developed to breakdown PET-based plastic bottles [174]. Further studies have been performed on PHAs, as it is more readily biodegradable in aerobic environments, and PLA (although it is shown to be resistant to microbial degradation).

## 7. Summary

Biodegradable biobased polymers are potential candidates for replacing traditional ones due to the consideration of pollution and sustainability. This review paper provides a comprehensive review of this important class of materials. Specifically, this review paper provided information on the current mechanical and thermal properties developments, the environmental effects, and the economic viability of transitioning towards biodegradable biobased polymers.

Current research in the mechanical and thermal properties has shown that metal oxide nanoparticles and compatibilizers, such as maleic anhydride, can potentially improve the mechanical strength of biodegradable biobased polymers. Furthermore, the methods of improving the thermal conductivity of biodegradable biobased polymers have been improved by using more energy-efficient methods. Freeze casting or magnetic squeeze, and ambient drying have been shown to make chitosan aerogels have more favorable thermal properties than petroleum-based polymers.

Research into the commercial potential, life-cycle assessment, and environmental effects of biodegradable biobased polymers illustrated the complexity of completely transitioning away from conventional petro-based polymers. Although the analysis of the ROI and NPV of PLA production showed to be very profitable compared to HDPE production, a complete transition is still a challenge due to the sheer amount of land, water, and energy required for such a transition to be fruitful. However, further development in developing second-generation polymers and improvements in mechanical recycling processes can assist in overcoming this challenge. Furthermore, improving the reusability of biopolymers through self-healing mechanisms (such as using Murexide salts) can help heal cracks and defects within the polymer, thus expanding its lifespan.

With research emerging on novel materials and a shift to a bioplastic-based economy, a big challenge still exists, completely abandoning petro-based plastics. Future research should focus on production processes, policy and legislation, improving reusability and recycling processes, reducing costs, and developing new materials. A few recommendations regarding possible research and development focuses are outlined in the following section.

## 8. Recommendations for Future Research

Through this review of biodegradable biobased polymers and their potentiality to become an alternative to petroleum-based polymers, we recommend below the future research directions:

### 8.1. Improving Biodegradation Processes

Due to the many restrictions in the degradation of most common polymers, improvements must be made to existing degradation processes. Most current research proves the anaerobic and aerobic degradation process by mixing the polymers with pro-oxidants or natural polymers to increase the degradation rate. Furthermore, UV rays, heat, and light have pretreated the polymer. However, there have been few studies and data collected on how much these methods have improved the degradation rates of most used polymers (such as PLA or polyglycolic acid). With a better understanding of the effects of pretreatment processes and pro-oxidants on the degradation rates of polymers, one can improve the efficiency of the degradation of common plastics.

### 8.2. Reducing Microplastics and Subsequent Impacts on Environments

One danger in the degradation of plastics is the formation of microplastics, which take longer to degrade. Although the early stages of degradation are fast, once they become microplastics, the degradation rate slows. The development of microplastics can cause many environmental issues, as they harm most living organisms. Because most common plastics used in packaging and plastic bottles are oxo-degradable plastics, further research must be conducted to improve the degradation process of these plastics to reduce the amount of microplastics in the environment.

### 8.3. Developing a Standardized Testing Procedure for Bioplastic Degradation

As the need for waste reduction and lowered plastic consumption rises over the next decade, a common testing procedure must be developed to produce the most consistent and metric results. Agreed, there are common standards among which the degradation tests are run, but few draw important comparisons even among the same material, which can be challenging. This leads to inefficient data reporting and collection, and cross-comparisons between studies are not nearly as accurate, which causes a wider range of results. For example, a 28-day period for compost-degrading bioplastic should be employed, and it should have a set temperature, environment, and moisture, among other things. Only one degradation measure should also be employed, such as CO_2_ evolution. CO_2_ evolution provides more accuracy, as there is a direct measure of how much plastic has been degraded. SEM could be a useful, more in-depth measure, but the degradation assessment is more subjective due to its multifaceted nature.

### 8.4. Analyzing Compost Compositions and Environments

Composting is one of the most effective yet difficult methods of biodegradation for biopolymers. However, the compost’s temperature, moisture, and environment must be monitored very closely for degradation to take place for a lot of bioplastics due to the nature of the microbial activity that occurs. Especially considering PLA, these factors must be given attention; otherwise, it could result in no mass change. Throughout many of the studies presented, some plastics resulted in minimal to no degradation. Further research should be pursued into what compost composition and environments best suit a given bioplastic.

### 8.5. Developing an End-to-End Production Process

While the general appeal of bioplastics is that they can be degraded in nature and not cause environmental harm, an end-to-end production process that utilizes bioplastics’ byproducts, instead of letting it degrade, would be far more efficient. Facilities that accept used bioplastic products and convert them back into commercial-ready bioplastics and the optimization of its production processes could be revolutionary for the bioplastic industry. Despite decades of research, bioplastics have not been implemented in everyday plastic usage. A more efficient production and recycling method can potentially change this.

### 8.6. Identifying and Focusing on Materials with the Highest Potential

Based on viability and mechanical performance, certain materials, such as PLA, should be focused on more. PLA is one of the highest potential bioplastic materials because of how relatively easy and cheap it is to produce. Although PLA could degrade slower than some other materials in most environments, such as PHB or PCL, it has been developed more as a material, researched extensively, and produced on a larger scale. PLA-based and starch-based biopolymer materials should be emphasized further in the literature to make an initial shift to a larger bioplastic economy possible, with more realistic techno-commercial production models being developed for practical applications. Bioplastics have been written about and researched for multiple decades now; a shift toward bringing this to every consumer is the next step, and progressing this idea further with a few bioplastic materials with the highest potential would allow for the idea to become closer to turning into a reality.

## Figures and Tables

**Table 1:** Comparison of direct and indirect costs for case one and case two (Table created using data from Ref. [61].

	PLA Production Case 1 (Million USD)	PLA Production Case 2 (Million USD)	HDPE Production (Million USD)
**Direct Cost** - Operating Cost	225	246	77
- Investment Cost	330.74	330.74	108.74
**Indirect Cost** - CH_4_ Emissions	20	10	13
- CO_2_ Emissions	10	10	124
- CO Emissions	-	-	1
- SO_2_ Emissions	-	-	3
- NO_2_ Emissions	-	-	2
- Opportunity Cost of Land	0.92	0.92	0.92
- Total Indirect Costs	30.92	20.92	143.92
**Total Cost**	586.66	597.66	329.66
**Benefits** - Direct Benefits	300	300	143
- Indirect Benefits	294	-	-
**Total Benefits**	594	300	143
**Net Benefits**	7.34	−297.66	−186.66
